# The Impacts of Transportation Infrastructure on Sustainable Development: Emerging Trends and Challenges

**DOI:** 10.3390/ijerph15061172

**Published:** 2018-06-05

**Authors:** Luqi Wang, Xiaolong Xue, Zebin Zhao, Zeyu Wang

**Affiliations:** 1 School of Management, Harbin Institute of Technology, Harbin 150001, China; luqiwang@hit.edu.cn (L.W.); xlxue@hit.edu.cn (X.X.); 2 School of Management, Guangzhou University, Guangzhou 510006, China; wangzeyu@gzhu.edu.cn

**Keywords:** transportation infrastructure, sustainable development, scientometric analysis, visual analysis, collaboration network

## Abstract

Transportation infrastructure has an enormous impact on sustainable development. To identify multiple impacts of transportation infrastructure and show emerging trends and challenges, this paper presents a scientometric review based on 2543 published articles from 2000 to 2017 through co-author, co-occurring and co-citation analysis. In addition, the hierarchy of key concepts was analyzed to show emerging research objects, methods and levels according to the clustering information, which includes title, keyword and abstract. The results expressed by visual graphs compared high-impact authors, collaborative relationships among institutions in developed and developing countries. In addition, representative research issues related to the economy, society and environment were identified such as cost overrun, spatial economy, prioritizing structure, local development and land value. Additionally, two future directions, integrated research of various effects and structure analysis of transportation network, are recommended. The findings of this study provide researchers and practitioners with an in-depth understanding of transportation infrastructure’s impacts on sustainable development by visual expression.

## 1. Introduction

Transportation infrastructure, as a complex network, connects cities and accommodates human activities coupling the social, economic and environmental systems with the urbanization and population growth. Additionally, the transportation network contributes to the socioeconomic development and the increased quality of life through generating inter- or intra-city connections during urbanization [1,2]. In addition, goals such as low-carbon, resilient and sustainable development should not be ignored when the transportation network is expanded [3]. In detail, transportation infrastructure among cities leads to urban aggregation and diffusion, greatly boosting the regional and national economic development [4,5]. However, the irrational planning of transportation infrastructure also generates negative effects, such as the ecological destruction, increased traffic accidents, climate change, CO_2_ emissions and lower transport efficiency [6,7,8,9,10,11]. Therefore, it is necessary to identify multiple impacts of transportation infrastructure from existing studies.

Recently, the impact of transportation infrastructure has been a hot topic, and the economic effect of transportation infrastructure has been receiving more attention and debate [12] because of the pursuit to direct economic growth of both regions and sectors [13]. To review multiple impacts of transportation infrastructure, scientometric studies have been used to analyze the literature and reveal trends in some specific topics such as transport phenomenon [14] and public transport [15]. However, in the field of transportation, existing scientometric studies mainly focus on statistical results, lacking the exploration of visual and network structure analysis. Therefore, this paper analyzed the co-author, co-occurring and co-citation network based on the collected literature expressed by visual graphs. The software Citespace was used to build the author and literature collaboration network and the co-citation analysis based on the expanded data from the citation dimension. This expansion increases the potential data source and improves the accuracy of review analysis. More importantly, scientometric study based on network visualization is an effective way to identify representative researches in the network structure and find the phenomenon and regularity compared with traditional literature analysis.

In this paper, we present a scientometric and systematic review that explores the literature related to the impact of transportation infrastructure in the database of Web of Science from 2000 to 2017. The aims of this study are identifying the research trends in the field of the transportation infrastructure and finding the hot research topics through the visualization map built by the literature. This paper is divided into three main parts. Section 2 introduces the basic concepts, characteristics and multiple impacts of the transportation. Theoretical analysis provides an in-depth understanding of the impact mechanism of transportation infrastructure according to existing studies. Section 3 introduces the scientometric method in this paper. This method provides a means of visualization to identify the information in the map based on the software Citespace. Section 4 analyzes the scientometric results, including co-author, co-occurring and co-citation analysis. Finally, according to the identified cluster data, this paper systematically summarizes representative studies and important categories related to the effect of transportation infrastructure. Multiple analysis greatly increases the accuracy of the results.

## 2. Transportation Infrastructure

### 2.1. The Definition and Characteristics of Transportation Infrastructure

As one of the main urban elements, transportation infrastructures such as roads, highways, railways, airports, bridges, waterways, canals and terminals play important roles in the transmission of materials and the flow of population during urban agglomeration and diffusion [16,17,18]. Just as stated in the definition given by OECD (2013), transportation infrastructure is a critical ingredient in the economic development at all levels of the income, supporting personal well-being and economic growth. From the perspective of function, transportation infrastructure is a kind of large-scale public work which has the importation influence on countries’ politics, economy, society, science, technology development, environmental protection, public health and national security. Besides, as a part of transportation system apart from the operating system and transport vehicles, the plan and construction of transportation infrastructure are complex. Grimsey and Lewis think it is easier and more meaningful to identify infrastructure than to define the infrastructure, and the key to identifying the infrastructure is indicating its characteristics [19]. For example, during construction, it has characteristics of large investment scale, long construction period, complicated risk, and many stakeholders [20].

Transportation infrastructure has the fundamental features of general infrastructure, such as high risk, high investment, complex organization and low income [21]. Additionally, it has another two special characteristics: geographic network and spatial externality [22]. On the one hand, transport infrastructure is a network infrastructure that constitutes the channel between nodes, regions or node-region. This promotes the spatial transfer of production factors and mobility of goods. On the other hand, the externality means that positive or negative effects on external subjects are generated when one economic entity produces or consumes. In terms of positive externalities, transport infrastructure as a public investment could directly promote economic growth and also indirectly increase the economy through spillover effects such as knowledge spillover effect and technology spillover effect. Meanwhile, environmental pollution and urban noise often happen because of the building of transport infrastructures, driving the generation of negative spillover effects. The existence of complex characteristics and significant roles drives the generation of multiple impacts of transportation infrastructures on the economy, society and environment.

### 2.2. The Multiple Impact of Transportation Infrastructure

The transportation infrastructure represents the motivator of economic growth and social welfare [23] through improving production performances and investment performances for the private sectors [24]. More specifically, the construction of transportation infrastructure could reduce the travel cost, attract foreign investment and expand trade of shared resources [25]. In terms of the social overhead capital, transport infrastructure plays a decisive role in industrialization and has obvious spillover effects on regional innovation, factor reallocation and manufacturing productivity [26], which promote the aggregation of industries, population and economy [16]; this is often called the economic distributional effect. However, some empirical studies have shown that the expansion of high-speed railway networks promotes the development of central cities but causes the economic growth rate of prefecture-level cities along the rail line to decline, which is referred to as the siphon effect [27]. Although different results were found based on various data sources or research objects, the empirical study is the most common and effective method to identify the positive or negative effects of transportation infrastructures.

Meanwhile, excessive infrastructure construction could put huge pressure on the natural and ecological environment when meeting the need for economic development and social improvement [28]. Transportation infrastructure provides the fundamental conditions for economic activities, while some spillover effects happen concomitantly [29], such as CO_2_ emission generated via domestic and global production networks [30], ecological destruction because of the biological habitat fragmentation [11] and the change of water flow and declining water quality [31,32]. Since the United States published the Environmental Impact Assessment (EIA) in 1969 [33], environmental problems have become a significant part of the law, and many topics have received wide attention. For the transport sector, apart from cost-benefit, design and investment analyses, environmental impacts such as CO_2_ emission and air quality are the main evaluation criteria [34]. In addition, some universal and systematic methods have been used to evaluate environmental performances, such as the multi-criteria model, meta-analysis [35], ecological footprint index [36], and value equilibrium analysis [37]. From the perspective of the environment, the effects of the transportation infrastructure are almost all negative, so minimizing the environmental impact has been the main research topic. Additionally, transport infrastructure assumes important social responsibility [38,39]. Although more jobs and optimized income distribution occurs after huge capital investments in infrastructure projects, health hazards, land expropriation and wildlife damage problems should not be neglected.

The multiple impacts of transportation infrastructures have received huge attention. However, the economic externality is still the most important and popular topic, which often ignores the environmental and social aspects [40]. Since the sustainable development topic has been a point of focus, the sustainable evaluation of transportation infrastructure has been increasingly valued. Based on the traditional cost-duration-quality decision model [41], plenty of indicators and methods have been extended to identify and assess transportation sustainability. For example, some multi-criteria models based on panel data have been extended, such as the multivariate co-integration approach [23], fuzzy logic evaluation [42] and the decoupling model [43]. In addition, optimizing the network structure and analyzing the spatial relationships of infrastructure operation are the key ways to promote the urban sustainability [44]. The complex characteristics and multiple impacts of transportation infrastructures have promoted studies on the identification and modelling of transportation sustainability. However, existing studies have mainly depended on experience to review the published articles. In addition, systematic and scientometric analysis could show complete and clear research status in this field.

## 3. Method

In the field of transportation, many reviews have been published to identify the research status, while most reviews have mainly depended on researchers’ backgrounds and experiences. To build an overview of existing studies with a relatively complete literature, the scientometrics method was used to find out the scientific regularity related to the effects of transportation research based on mathematical statistics and computing techniques [45]. In addition, scientometric analysis mainly depends on bibliographic data to identify the research trends and literature relationships [46]. The scientometric method was used in this study to build a visual information graph for further data mining using the software Citespace (http://cluster.cis.drexel.edu/~cchen/citespace/). The visualization process of the bibliography is meaningful for discovering the potential information based on the graphical representation of data using shapes, colors and images [47]. This method reduces the difficulty in analyzing a large literature, and effectively finds the regularity and the hidden information in existing studies. In this section, the data overview and research path of the scientometric method are presented.

### 3.1. Data Overview

The Web of Science (WOS) database was used to collect published literature data related to the transportation infrastructure. Apart from WOS, Google Scholar’s database is extensive, but its citation information is incomplete and inconsistent [48]. Therefore, it is difficult to use for scientometric analysis. In addition, WOS contains the most important and influential journals in the world [49,50]. The impact of transportation infrastructure includes many categories, such as human, economic and environmental. Therefore, in this section, a comprehensive data overview is presented to show the trend of existing studies. In addition, “transport infrastructure” and “transportation infrastructure” were used as keywords to collect the data, initially.

**Figure 1:**
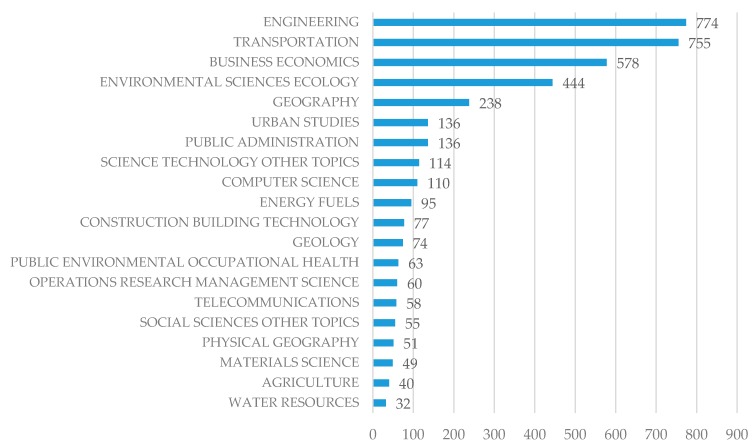
The top 20 research fields of the transportation infrastructure.

This paper analyzed all collected literature in the WOS core database from 2000 to 2017. The search code SS = (transport* infrastructure*) was used in the WOS core collection. Here, “*” denotes a fuzzy search and “SS” means an article subject search. A total of 2543 bibliographic records were collected in October, 2017, and there are 14 related records filtered by being highly cited in the field, as shown in Table 1. Highly cited papers are the top one percent in each of the 22 Essential Science Indicators (ESI) subject areas per year, which indicates scientific excellence. We can see that this literature is distributed over recent years, and almost all records are related to the environmental dimensions. It is notable that the highest cited article was published in 2012 and is about biofuel application in transportation vehicles. Additionally, Figure 1 shows the top 20 research fields related to transportation infrastructure, including engineering, transportation, business economics, environmental sciences, computer science, geography, public administration, urban studies, and so on. This means the studies related to transportation infrastructure range from the technological level to the management level, providing more challenges and opportunities to interdisciplinary research.

**Figure 2:**
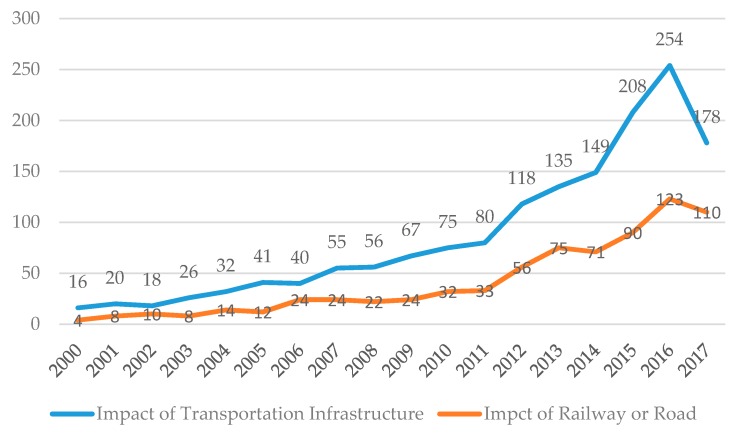
The number of articles on impact of transportation infrastructure.

The data overview above shows the overall research trends and fields. According to the research scope and objects, some keywords are chosen to filter the results that are more related to the spillover effects of the transportation infrastructure network. Then the words (SS’ = effect* or affect* or influence* or impact*) were selected to refine the results, and a total of 1568 bibliographic records were searched. This step refined the records referring to the impact of transportation infrastructures or other effects on transportation infrastructure. Finally, the main keywords (SS’’ = railway* or rail* or road* or highway* or expressway* or freeway*) were used to further refine these results in accordance with the specific research objects of this paper, and got 764 records. Figure 2 shows the distribution of 1568 bibliographic records related to the two-way influence of transportation infrastructure and the records related to the influence of railway or road. In addition, the final 531 papers were used for further review analysis. Multi-step data filtering benefits a narrow data range, promoting study depth and guaranteeing the data integrity. It is clear that the distribution trend of is similar between the original data and the filtered data, which means the impact of railway and road could follow the path of the development of transportation infrastructure. In addition, research about the impact of railways and roads accounts for around 20–40% of research on transportation infrastructure during the timespan. In other words, the analysis of railways and roads partially represents the transportation infrastructure.

### 3.2. Scientometric Method

Scientometric analysis is a systematic method to identify and analyze the published literature, and it has become increasingly frequently used to obtain a deeper understanding of a research area [51]. In addition, this analysis has been recognized as an efficient method to identify the hidden information in published bibliographies [52]. In the field of transportation, scientometric analysis has been used as a quantitative approach to identify research phenomena and trends [14,15], but these previous studies did not systematically analyze the research network or recognize the hidden research trends and relations. The software CiteSpace can visualize the emerging trends, transient patterns, substantial theoretical and methodological contributions in scientific literature from the perspective of a social network [53,54]. The accessible graphs based on network analysis and clustering algorithms are able to show the knowledge more logically and systematically [55]. Therefore, CiteSpace was used to identify and analyze the main effects of transportation infrastructure on sustainable development based on the literature. In this study, some scientometric techniques were used, such as fundamental information analysis (author, institution and country) and network analysis (subject, keywords and co-citation). According to these analysis results, the research challenges and trends were further systematically analyzed.

**Figure 3:**
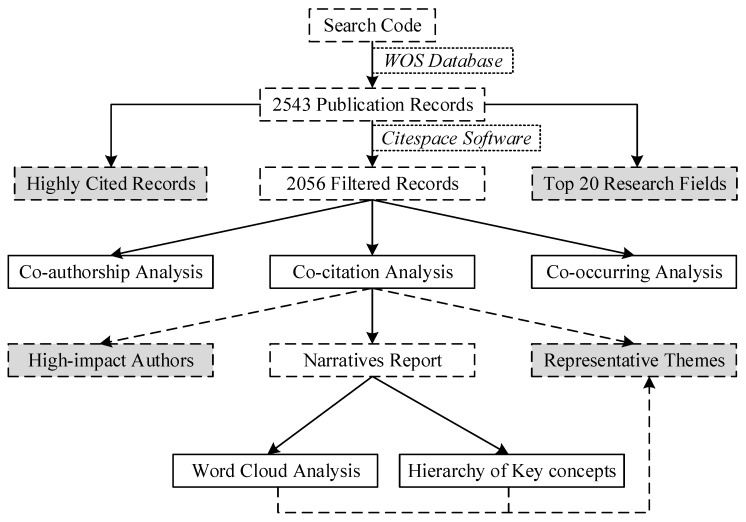
Research procedure of this study.

In detail, the research procedure of this study includes three main parts, according to the collected bibliographic data, as shown in Figure 3. Firstly, 2543 records were collected to perform the data overview, including the highly cited analysis and the top 20 research fields. After the filtering, 2056 records were analyzed by CiteSpace software to show representative people, institutions, countries and relationships among them. Then the dual-map overlay and keyword network of the literature were analyzed to show representative research subjects and issues. Additionally, references in the collected literature were analyzed to build the co-citation network, which generates the clustering information to expand the data source. Finally, according to the clustered information, the research status and trend were summarized systematically to generate the hierarchy of key concepts. All of these steps reviewed the bibliographic information from different dimensions to find the respective research issues.

## 4. Results and Discussion

### 4.1. Co-Authorship Analysis

**Figure 4:**
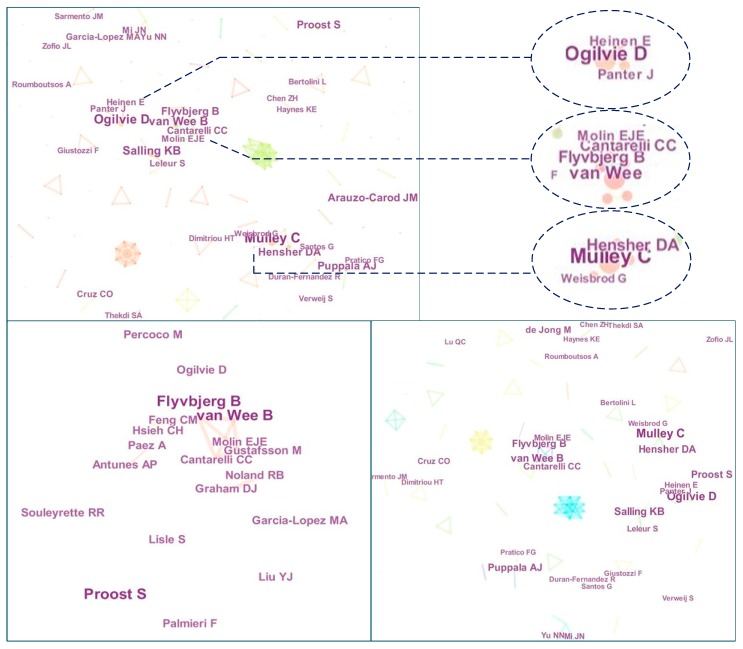
Comparison of author collaboration networks.

According to the author collaboration analysis, the domain authors have a relatively large number of links to other authors in the network, which means the domain authors have higher academic relevance [56]. In this study, 2056 valid bibliographic records were collected from 2000 to 2017. The co-authorship network is shown in Figure 4, where each node represents an author and links between authors denote collaboration established through co-authorship of articles. In this network, excessive links were removed by Pathfinder using network pruning [57], and eventually 189 nodes and 173 links were identified. In addition, the node size represents the frequency of published references and the node color accounts for different collaboration modularity.

According to the cluster information from 2000 to 2017, the network density is 0.0097 and the cluster modularity Q is 0.8626, which means the network of co-authors is fragmented. Only a few closed circuits exist in the network, such as the Mulley C group, Flyvbjerg B group and Ogilvie D group. As shown in the left-bottom graph of Figure 4, many authors collaborated with one or two productive authors. For example, Flyvbjerg Band Van Wee B were both productive and central authors in the community. All centralities of these groups are small, which indicates that in the timespan 2000–2010, important collaboration groups were not formed by author centrality. In order to determine the timeliness of the study, the research period was limited to 2010–2017. In addition, the right-bottom graph of Figure 4 shows the author collaboration network during this period. In this network, 1899 valid records are included and there are 185 nodes and 172 links. The network density is 0.0101, which is similar to the left graph. The network modularity and network structure only change slightly. It is notable that the author clusters change slightly, which means these central authors play significant roles in this field. Overall, from the perspective of timespan, author collaboration groups remained stable and relatively separate from the increased cumulative number of works in the published literature.

Apart from the collaboration analysis, author productivity is an important criterion to show the roles of authors or teams. Based on the 2056 collected bibliographic records, the top 10 most productive authors were identified in Table 2. This shows that the main research fields of the hot authors include transportation, business economics and environmental management. More importantly, the collaboration links among most productive authors were more frequent and the productive authors generally led to modularity. For example, the productive author Flyvbjerg Bent cooperated with another productive author Van Wee Bert and the productive authors Ogilvie David, Flyvbjerg Bent and Mulley Corinne generated co-author modularity, as shown in Figure 4, which means that the productive authors were often cited and focused upon.

### 4.2. Co-Author’s Institution and Country Analysis

**Figure 5:**
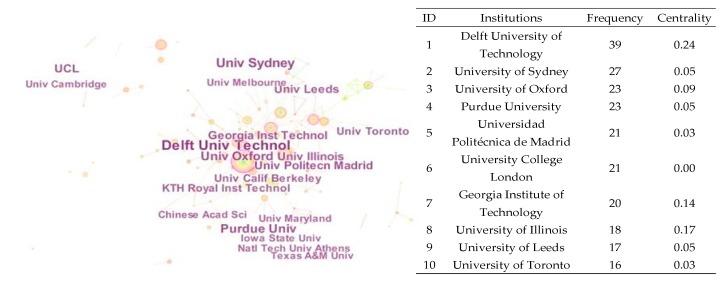
Institution collaboration network.

As shown in Figure 5, the institution network includes 280 nodes and 236 links from 2000 to 2017. The node size represents the amount of published literature from one institution. According to the collected information, the studies related to transportation infrastructure were rich at institutions such as Delft University of Technology (50 records), University of Sydney (33 records), Universidad Politécnica de Madrid (29 records), University College London (28 records), University of Oxford (28 records) and Chinese Academy of Sciences (25 records). This indicates that transportation infrastructure research was active and advanced. In addition, institution nodes with high betweenness centrality are shown in Figure 5. The size of the colored circle represents the amount of published literature in one institution, and different colors show the number in different years. Institutions with high centrality play important roles in the institution network, such as Delft University of Technology (centrality = 0.24), University of Illinois (centrality = 0.17), Georgia Institute of Technology (centrality = 0.14), Shanghai Jiao Tong University (centrality = 0.12) and University of Florida (centrality = 0.10), and they drove the research collaborations among different institutions. Apart from the Delft University of Technology, the top productive institutions did not have higher relative centrality. This means that institutions that published more articles did not play an equally important role in the collaboration network. The institutions with higher centrality would have greater potential.

**Figure 6:**
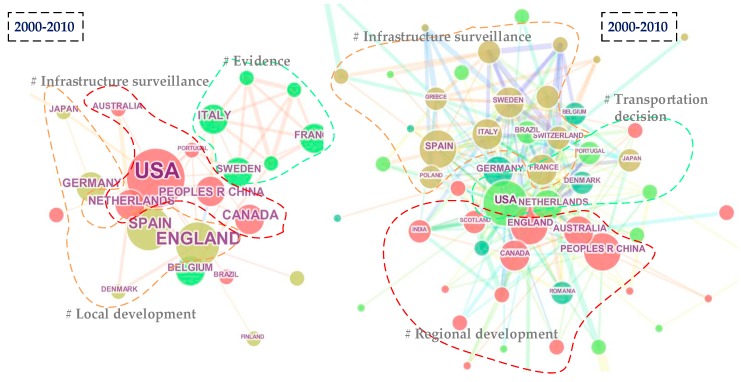
Comparison of country collaboration network.

Furthermore, Figure 6 shows the country collaboration network in 2000–2010 and 2010–2017; clusters are displayed in different colored circles and they are arranged vertically in the order of their size. In addition, the colored lines represent co-citation links among different countries. During 2000 to 2010, as shown in the left graph, the top 5 countries with the highest centrality include USA (centrality = 0.65), England (centrality = 0.4), Sweden (centrality = 0.36), Italy (centrality = 0.21) and Japan (centrality = 0.14), which means that they occupied key positions in the collaboration network. During 2010 to 2017, as shown in the right graph, the top 5 countries with the highest centrality include USA (centrality = 0.53), England (centrality = 0.31), Germany (centrality = 0.17), Australia (centrality = 0.15) and the People’s Republic of China (centrality = 0.1). We can see the centralities of USA and England experienced a decrease and the roles of Germany, Australia and the People’s Republic of China became increasingly significant. Additionally, apart from the top central countries, Spain, Netherlands and Canada had higher published frequencies, which indicates their higher relative potentials. According to the clustering results, we can see the change of research interests. The labels of clusters were generated by log-likelihood ratio method in the software. It is notable that during 2000–2010, an overview of popular topics included infrastructure surveillance, local development and evidence; during 2010–2017 those consisted of transportation decision, regional development and infrastructure surveillance. In addition, the clustering members experienced an increase and transfer.

### 4.3. Co-Occurring ANALYSIS

#### 4.3.1. Discipline Analysis

**Figure 7:**
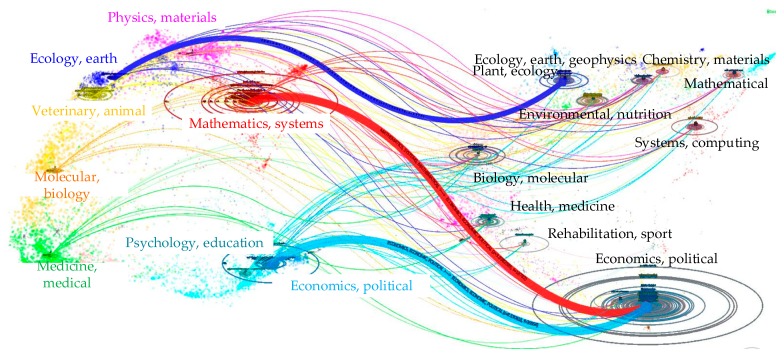
A dual-map overlay of the literature’s journals.

**Figure 8:**
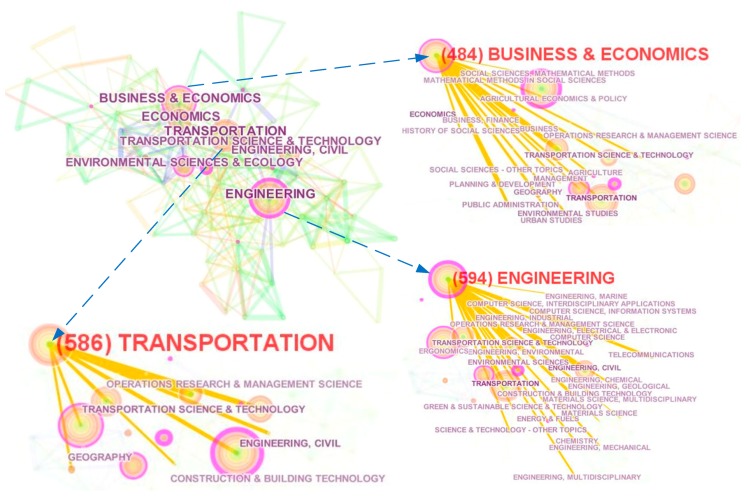
Inter-disciplinary co-occurring network of the literature.

Every citation and cited work was assigned to a specific research discipline according to the journals in a global map of science generated from over 10,000 journals indexed in the WOS [58]. Therefore, this study built an overlay map to show the dual-map of the science sketch database that perfectly described the interdisciplinary research. Figure 7 shows the main disciplines of collected citing articles and cited articles. The left part of the graph shows the distributed disciplines of citing articles and the right part describes that of cited articles. In addition, the color curves represent the fluctuant relations. It is clear that the journals of citing articles related to transportation infrastructure are mainly distributed in disciplines such as mathematics, systems, economics and physics. Cited articles’ journals are mainly distributed in the areas of ecology, computer, social education and economics. The distribution of cited articles indicates the application fields and research foundations. More importantly, transportation infrastructure papers are published in almost all major disciplines, which means transportation infrastructure studies play important roles in multidisciplinary research. Additionally, the dual-map overlay shows the information about the field studies more macro compared with article clustering analysis. Thus, Figure 8 shows the interdisciplinary co-occurring network of the literature based on the WOS discipline categories. We can see that the top frequent disciplines include Engineering, Transportation and Business & Economics. The links among different nodes mean the existence of collaboration among different disciplines. Interdisciplinary research is quite obvious in the field of transportation infrastructure.

#### 4.3.2. Co-Occurring Keyword Analysis

**Figure 9:**
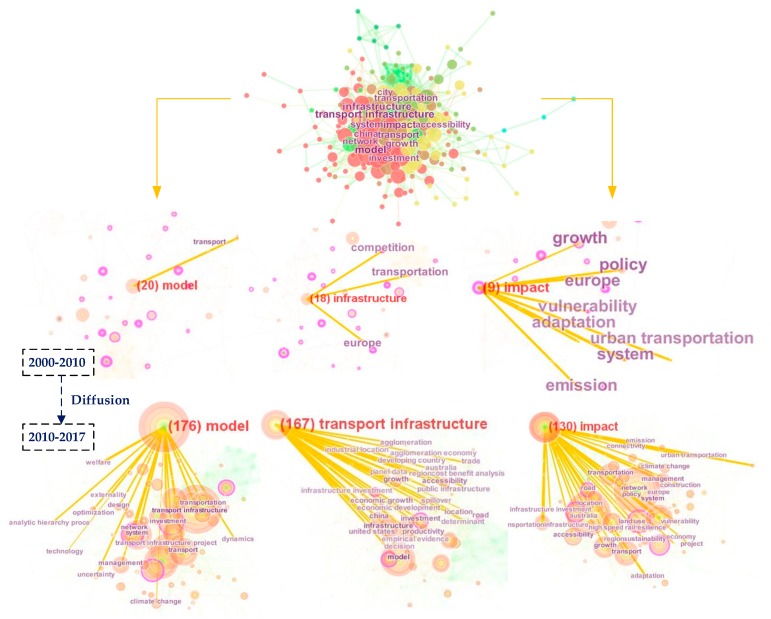
Keyword co-occurring network diffusion.

Keywords catch the core content of a paper, and in this section, the collected keywords show the situation and development of research using the software CiteSpace. According to the 2056 valid records collected, the keyword co-occurring network includes 225 nodes and 1092 links shown in Figure 9. The node size represents the frequency of a keyword in all records and links among nodes indicate different keywords occurring in the same record. The t-SNE view was used to lay out the keyword map. The t-SNE technique is a perfect visual method for this map, and gave a complete and clear description. Among the top 50 hot keywords, the most frequent keywords include model (frequency = 176), impact (frequency = 120), system (frequency = 109), growth (frequency = 94), investment (frequency = 86), network (frequency = 85), accessibility (frequency = 80), city (frequency = 74) and policy (frequency = 72), in addition to transportation infrastructure (frequency = 225). More importantly, China (frequency = 86) and the United States (frequency = 52) are two representative country keywords, which means that in these two countries, studies related to transportation infrastructure attracted more attention. In addition, some keywords with high frequency had a relatively high centrality, such as city (centrality = 0.15), network (centrality = 0.14), investment (centrality = 0.12) and impact (centrality = 0.09). To indicate the change of hot topics, we divided the timespan into 2010–2010 and 2010–2017, as shown in Figure 9. The top three keywords are model, infrastructure and impact. The related keywords experienced a significant increase; in particular, keyword impact-related topics included climate, urban studies, land use, resilience and accessibility, which indicated this role. However, this network only shows information based on the collected records, and its difference from the co-citation network is the limitation of this relatively incomplete data. Therefore, the co-citation analysis further solves the data incompleteness in the next section.

### 4.4. Co-Citation Analysis

**Figure 10:**
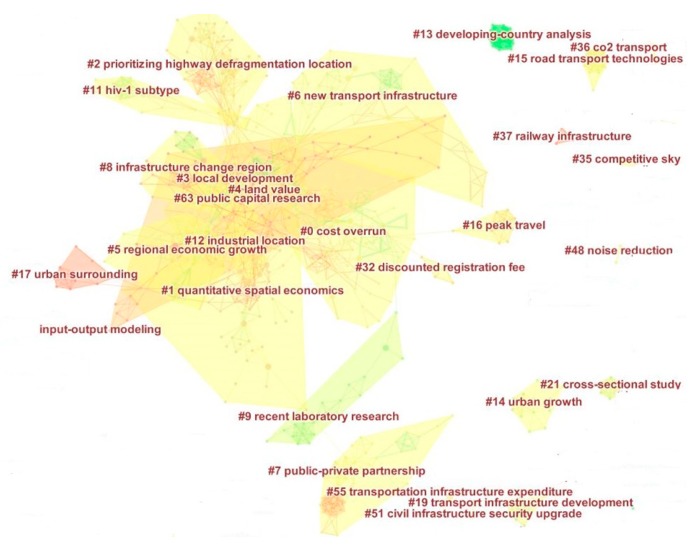
A landscape view of the co-citation network.

Co-citation analysis has been defined as the frequency with which two articles are cited together in another article [59]. In this section, co-citation analysis identifies the underlying intellectual structures of the knowledge in the field of transportation infrastructure according to references. The co-citation network was generated based on 2047 valid records between 2000 and 2017, and the top 50 most cited publications in each year were used to construct a network of references cited in that year. As shown in Figure 10, the synthesized network contains 879 references and 174 co-citation clusters after the clustering process. This network has a modularity of 0.8934, which is considered to be very high, suggesting that the specialties in science mapping are clearly defined in terms of co-citation clusters. The mean silhouette is 0.3855, which is relatively low, mainly because of the numerous small clusters. The major clusters that we focus on in this paper were sufficiently high. The areas in different colors indicate the time at which co-citation links in those areas appeared for the first time. Areas in green were generated earlier than areas in yellow. Each cluster can be labeled by title terms, keywords, and abstract terms of articles citing the cluster. We can see that studies related to new application, cost overruns and case study appeared earlier, and urban transportation and public-private partnerships appeared more recently. In addition, cluster areas of new transport infrastructure, cost overruns and evidence study are relatively bigger, which means that these studies received more attention. According to the LLR, labels of the largest 62 clusters were summarized as shown in Appendix A and the most active citer can be checked in Appendix B.

**Figure 11:**
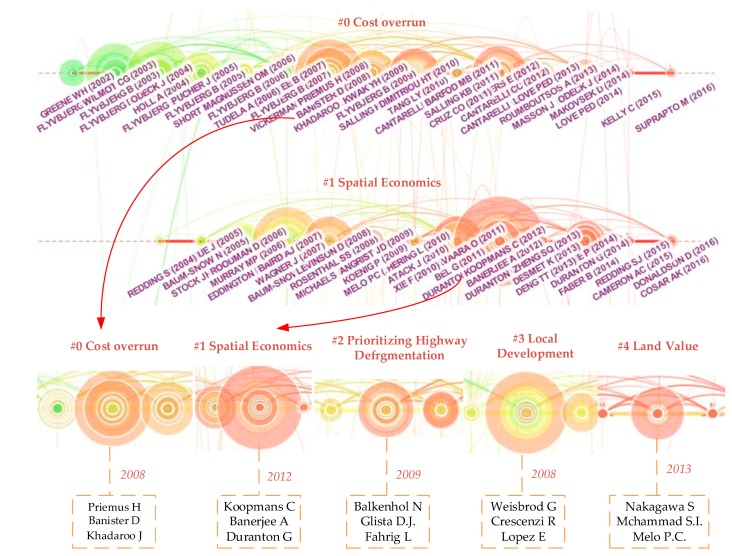
A timeline visualization of the largest clusters.

In addition, the timeline visualization in CiteSpace depicted clusters along horizontal timelines. As shown in Figure 11, each cluster was displayed from left to right and clusters were arranged vertically in descending order of their size. The colored curves represent co-citation links added in the year of the corresponding color. Large-sized nodes or nodes with red tree rings received particular attention because they were either highly cited or had citation bursts, or both. We can see that the three most-cited references in a particular year are displayed. The labels of these references were placed in the lowest position. The cluster labels were generated based on terms identified by Latent Semantic Indexing (LSI) [60]. Figure 11 shows the top 2 largest clusters, listed as cluster#0 and cluster#1. The periods in which the clusters were sustained were different, which means that the difference of topic activity. For example, topic #0 (cost overrun) was active during the period from 2008 to 2017 and most of the top active topics were active about 20 years. Furthermore, the top ten largest clusters include cost overrun, quantitative spatial economics, prioritizing highway defragmentation location, local development, land value, regional economic growth, new transportation infrastructure, public-private partnerships, infrastructure change region, recent laboratory research and microbial engineering. All of these clusters have relative network sub-structures and research status, and trends hide in these references. For example, for the cluster around spatial economics, 2011 to 2012 was the most active timespan for citers.

The analysis above shows the research base and fronts that mine the potential research challenges and trends. In addition, main research topics were further analyzed according to the selected and filtered data above. Table 3 shows the temporal properties of major clusters. We can see that most of the representative references are related to the spillover effect of the transportation infrastructure. For example, Cluster #0 (cost overrun) is the largest cluster, containing 94 references from 2011 to 2017. The mean year of all references is 2008 and the year of the most representative cited articles in this cluster is 2008, too. The timeline visualization reveals the top three cited references from the period of 2000 to 2017. As shown in Figure 11, the three most representative cited references (Priemus Hugo, Banister David and Khadaroo Jameel) occur in 2008. We can see that the period 2008 to 2016 was full of high-impact contributions—large colored citation circles and red citation bursts. We chose the top three cited circles and nine references to analyze the main research topics. Similarly, in the other five clusters, the top three circles and nine representative references were chosen to further analyze the hot research status and research trends. Appendix C shows the high-impact members of the other clusters. These authors may be not the most highly cited authors, but they play important roles in the corresponding fields.

### 4.5. Hierarchy Analysis of Key Concepts

**Figure 12:**
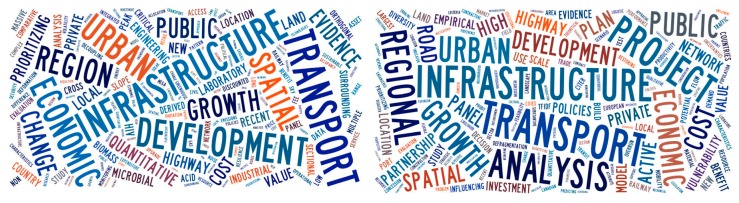
Word cloud distribution of co-citation cluster results. (Tool: Tagxedo (www.tagxedo.com). Data source: Labels of the top 62 clusters, narrative summary report of the co-citation network.)

The co-citation network above was divided into 174 co-citation clusters. These clusters were labeled by index terms from their own citers. These keywords show the most representative research topics related to transportation infrastructure. The left part of Figure 12 shows the word cloud based on cluster labels filtered by the same or similar labels of clusters. In this figure, the keyword size represents the frequency of cluster labels. It is clear that the main research topics include economic, region or urban development and spatial effect analysis. However, the cluster data only analyzed the label information, and did not identify other potentially relevant information. Therefore, a report of automatically generated narratives was used to analyze the word cloud distribution further, as shown in the right part of Figure 12. The narratives include the main subjects in the titles and abstracts of the top references in the top 62 clusters that are relatively complete. We can see that hot research topics consist of urban development, project, economic, cost and policies. In particular, we identified some potential topics that were excluded in the left graph, such as land, risk, panel data and policies. By means of the two-step summary, potential keywords could be easily identified.

**Figure 13:**
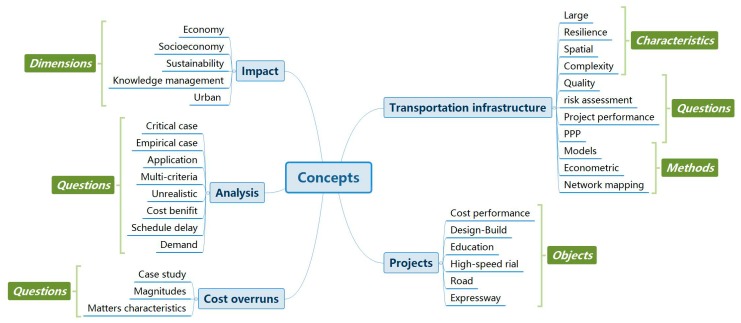
A hierarchy of key concepts in Cluster #0.

Additionally, key concepts identified from the titles of citing articles in Cluster #0 were algorithmically organized according to hierarchical relations derived from co-occurring concepts. Figure 13 shows the main concept tree of Cluster #0. The largest branch of such a hierarchy typically reflects the main concepts of scholarly publications produced by the specialty behind the cluster. The main logical categories include transport infrastructure, projects, cost overruns and impact. The category “Transportation Infrastructure” mainly focuses on improving the project performance separately from the traditional and important problem “Cost Overrun”. In particular, the category “impact” emerged gradually as an independent branch, which was driven by the increasing quantity and complexity of transportation infrastructure. It is notable that the transportation infrastructure branch highlights the characteristics (large, resilience, spatial and complexity), research methods (modeling, econometric and network mapping) and research questions (quality, risk, performance and PPP). In other words, sub-categories in this figure indicate the characteristics, questions, objects, dimensions and methods related to transportation infrastructure. The identity of category labels based on the title data obeys the logical tree algorithm of the software Citespace. This figure not only shows the main research topics but the logical relationships among these topics.

**Figure 14:**
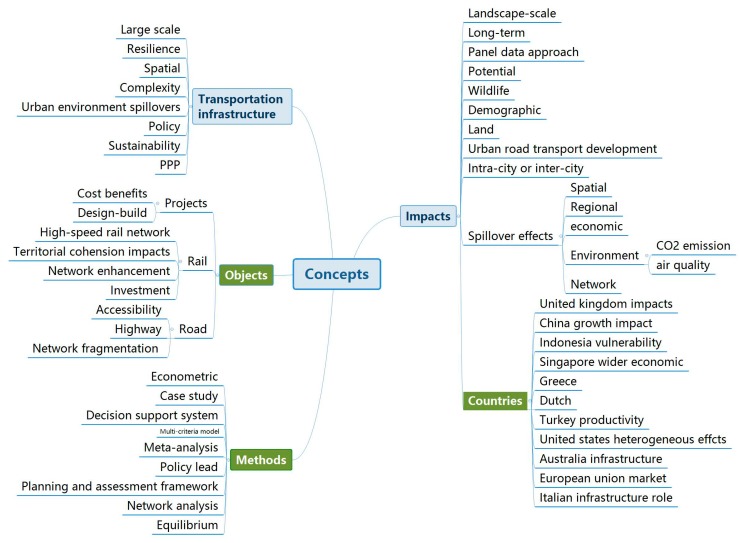
A hierarchy of key concepts in Cluster #0–#6.

To understand the hierarchy better, the key concepts in the top 7 Clusters (#0–#6) were identified in one hierarchy. Figure 14 summarizes the concept tree generated by Citespace according to the reference titles in Cluster #0–#6. The categories colored blue were identified automatically. For the systematic expression of the hierarchy, some branches colored green are used to conclude the fragmented research questions. In addition, this hierarchy filtered the repeating keywords and deleted words which cannot indicate the main research questions, such as “analysis”. However, the sub-category concepts of the branch “analysis” were distributed in other branches. Meanwhile, some sub-categories of the branch “transportation infrastructure” were distributed in the summative branches such as the “objects” and “methods”. We can see from Figure 14 that the main branch is “impacts”, in which the “spillover effects” and “countries” are listed separately. This means the topic of spillover effect is the intensive research issue, and there are many countries analyzing the impacts of transportation infrastructure on the national scale. In addition, the “impact” category summarized some detailed topics such as land use, urban development and spatial effect. Compared with Figure 13, this hierarchy identified more specific topics, such as rail and road research. Although the amount of data in Figure 14 is about seven times greater than in Figure 13, the hierarchy framework becomes more clear and systematic after filtering out repeated data. More importantly, this systematic hierarchy can help to identify the hottest and most representative research issues quickly.

## 5. Conclusions

This scientometric review based on over 2500 publications from 2000 to 2017 presented the systematic knowledge structure related to impacts of transportation infrastructure on sustainable development. Due to the complex impact mechanism, the identification process needs an in-depth understanding and clear expression. Although reviews related to transportation infrastructure have received attention, the scientometric review with visual expression provides a better way to explore the potential information hidden in knowledge network compared with the traditional review. In this paper, the presentation of scientometric and systematic reviews includes four main steps. Firstly, co-author analysis was used to identify the highly productive authors, institutions and countries to show the overall research status. Then, the co-occurring analysis was used to identify and visualize the overall research trends based on discipline and keyword information. Next, citing articles and cited references were modulated to find co-citation relationships and modularity labels by timeline visualization. Finally, after the modularity, the cluster information was analyzed further to conclude the hierarchy concepts of the main clusters, which accurately identified key points. These four steps analyzed the research status and emerging trends of transportation infrastructure’s impacts on sustainable development from multiple perspectives, such as author information, collaborative relationships and reference relationships. In addition, compared with the traditional literature review, this scientometric analysis shows the representative information clearly based on a visual map. Importantly, this visual expression provides an easier way to understand the complex collaboration network of literature.

The main research findings are as follows. First, collaboration links among the most productive authors were more frequent than other authors. Moreover, the productive authors generally led to modularity. Second, institutions with high centrality play important roles in the institution network, such as Delft University of Technology, University of Illinois and Georgia Institute of Technology. In addition, countries occupying key positions include USA, England and China. Third, the hot topics related to transportation infrastructure include cost, performance, quality and investment issues from the project level. In addition, from a more macro perspective, economic, social and environmental effects of transportation infrastructure were all caught. Fourth, according to the hierarchy analysis, specific research objects, methods and multiple effects of transportation infrastructure were identified. It is noticeable that spillover effects of transportation infrastructure include some dependent sub-categories, such as spatial, regional, economic and environmental effects. These more macro keywords indicate the complexity of impact mechanisms. In addition, transportation infrastructure has huge impacts on land, urban development, human life and city networks.

However, there are also some limitations that need further improvement in this study. A limitation of using bibliographic databases is that the WOS lacks the information of books and reports in public sources, thus necessitating the integration of multiple data resources. In addition, due to the limitations of the analysis tool, our study could not analyze the information hidden in the references’ context. Additionally, the determination of search keywords mainly relies on the subjective judgment of the authors, which might lead to data being missing or incomplete. Given these limitations, multiple analysis was exerted in this work to make up for the data limitations. In this study, the titles, keywords and abstracts could be credibly representative of the main context. Our findings not only reveal research trends, but future research directions. In the future, two directions—integrated study of various spillover effects and network effects of mega transportation infrastructures such as railway and road—will be valuable research issues. By conducting further research in these directions, an improved understanding of the significance of the transportation infrastructure will be obtained, and the planning of transport networks will be conducted under proper advice. In conclusion, this study provides valuable information for both researchers and practitioners to understand the significant and complex impact of transportation infrastructure. It is clear that in both technological issues and management issues, the impact assessment is the key step to justifying the research in the field of transportation infrastructure. This scientometric review will lead to the construction of a theoretical framework to guide this practice.

## Figures and Tables

**Table 1:** Top highly cited research categories.

Title	Cited	Year	Sustainable Dimensions		
			Economy	Environment	Society
Microbial engineering for the production of advanced biofuels	409	2012	✓	✓	
Grasping at the routes of biological invasions: a framework for integrating pathways into policy	361	2008		✓	
Catalytic routes for the conversion of biomass into liquid hydrocarbon transportation fuels	336	2011	✓	✓	
Liquid-phase chemical hydrogen storage materials	294	2012	✓	✓	
Plug-in Vehicles and Renewable Energy Sources for Cost and Emission Reductions	287	2011		✓	
Adults’ Sedentary Behavior Determinants and Interventions	269	2011			✓
From roadkill to road ecology: A review of the ecological effects of roads	259	2007		✓	
Urban green space, public health, and environmental justice: The challenge of making cities ‘just green enough’	203	2014		✓	✓
Urban sprawl in the Mediterranean? Patterns of growth and change in the Barcelona Metropolitan Region 1993–2000	170	2008			✓
Robust alternative technology choices are required in the paradigm shift from the current crude oil-reliant transport fuel platform to a sustainable, more flexible transport infrastructure.	162	2011	✓	✓	✓
Impacts of urbanization on urban structures and energy demand: What can we learn for urban energy planning and urbanization management?	101	2011		✓	✓
Route Infrastructure and the Risk of Injuries to Bicyclists: A Case-Crossover Study	66	2012			✓
Changes in mode of travel to work: a natural experimental study of new transport infrastructure	17	2015		✓	✓
Impact of New Transport Infrastructure on Walking, Cycling, and Physical Activity	12	2016		✓	✓

**Table 2:** Top ten most productive authors.

Author	Institution	Count	Research Field
Flyvbjerg Bent	University of Oxford	15	Transportation, Business Economics, Public Administration, Urban Studies, Environment Sciences Ecology, Geography
Mulley Corinne	University of Sydney	13	Transportation, Business Economics, Geography, Engineering, Environmental Sciences Ecology
De Jong Martin	Delft University of Technology	12	Transportation, Business Economics, Government Law, Public Administration, Geography
Ogilvie David	University of Cambridge	12	Public Environmental Occupational Health, Nutrition Dietetics, Physiology
Proost Stef	Katholieke Universiteit Leuven	12	Transportation, Business Economics, Engineering, Operations Research Management Science, Environmental Sciences Ecology, Geography
Salling Kim Bang	Technical University of Denmark	11	Transportation, Business Economics
Van Wee Bert	Delft University of Technology	11	Transportation, Business Economics
Durango-Cohen Pablo	Northwestern University	10	Engineering, Transportation, Business Economics, Operations Research Management Science
Hensher David A	University of New South Wales	10	Transportation, Business Economics, Engineering, Geography
Manuel Vassallo Jose	Universidad Politécnica de Madrid	10	Transportation, Engineering, Business Economics

**Table 3:** Temporal properties of the major 6 clusters.

Cluster	Size	Silhouette	Mean (Year)	Theme	Alternative Themes
0	96	0.886	2008	Cost overruns	Transportation infrastructure project
1	59	0.825	2010	Quantitative spatial economics	Infrastructure spatial framework
2	57	0.976	2009	Prioritizing highway defragmentation location	Impacts approach
3	55	0.762	2007	Local development	Public transport investment
4	54	0.91	2012	Land value	Property value
5	52	0.903	2008	Regional economic growth	Regional economic growth

## Appendix A

**ID**	**Label**	**ID**	**Label**	**ID**	**Label**
0	Cost overrun	21	Cross-sectional study	42	Forecasting infrastructure resilience
1	Quantitative spatial economics	22	Spatial structure	43	Price-difference approach
2	Prioritizing highway defragmentation	23	Critical transportation infrastructure	44	Sustainable development
3	Local development	24	Panel data evidence	45	Retail travel pattern
4	Land value	25	Massive non-orthogonal multiple access	46	Traveler characteristics
5	Regional economic growth	26	Low-volume roads	47	Operational effect
6	New transport infrastructure	27	Planning transport infrastructure	48	Noise reduction
7	Public-private partnership	28	Comparative evaluation	49	Changing transport system
8	Infrastructure change region	29	Transportation infrastructure asset	50	Mega region economic development
9	Recent laboratory research	30	Temporal effect	51	Civil infrastructure security upgrade
10	Microbial engineering	31	Infrastructure network	52	Political volatility
11	hiv-1 subtype	32	Discounted registration fee	53	Greenhouse gas emissions reduction
12	Industrial location	33	Environmental benefit	54	Spatiotemporal variation
11	Developing-country analysis	34	Service delivery	55	Transportation infrastructure expenditure
14	Urban growth	35	Competitive sky	56	Resource allocation
15	Road transport technologies	36	CO_2_ transport technologies	57	Transport infrastructure investment
16	Peak travel	37	Railway infrastructure	58	Transport policies
17	Urban surrounding	38	Long-range transportation	59	International evidence
18	biomass-derived acid	39	Decoupling urban transport	60	Occupancy model
19	Transport infrastructure development	40	Complex urban infrastructure deformation monitoring	61	Solar energy
20	transport infrastructure slope	41	Integrated approach	62	Public investment research

## Appendix B

**ID**	**Label**	**Most Active Citer**
0	Cost overrun	Cantarelli, C.C. (2010) cost overruns in large-scale transportation infrastructure projects: explanations and their theoretical embeddedness
1	Quantitative spatial economics	Redding, S.J. (2017) quantitative spatial economics
2	Prioritizing highway defragmentation	Gurrutxaga, M. (2014) prioritizing highway defragmentation locations for restoring landscape connectivity.
3	Local development	Gutierrez, J. (2010) using accessibility indicators and gis to assess spatial spillovers of transport infrastructure investment.
4	Land value	Brey, R. (2017) is the widespread use of urban land for cycling promotion policies cost effective? A cost-benefit analysis of the case of seville.
5	Regional economic growth	Deng, T.T. (2013) impacts of transport infrastructure on productivity and economic growth: recent advances and research challenges.
6	New transport infrastructure	Ogilvie, D. (2010) commuting and health in cambridge: a study of a ‘natural experiment’ in the provision of new transport infrastructure.
7	Public-private partnership	Sarmento, J.M. (2016) anatomy of public-private partnerships: their creation, financing and renegotiations.
8	Infrastructure change region	Chen, C.L. (2017) can transport infrastructure change regions’ economic fortunes? Some evidence from Europe and China.
9	Recent laboratory research	Butler, E. (2011) a review of recent laboratory research and commercial developments in fast pyrolysis and upgrading.
10	Microbial engineering	Peralta-Yahya, P.P. (2012) microbial engineering for the production of advanced biofuels.
11	hiv-1 subtype	Tatem, A.J. (2012) spatial accessibility and the spread of hiv-1 subtypes and recombinants.
12	Industrial location	Arauzo-Carod, J.M. (2013) location determinants of new firms: does skill level of human capital really matter?
11	Developing-country analysis	Fare, R. (2009) optimal investment in transportation infrastructure when middlemen have market power: a developing-country analysis.
14	Urban growth	Aljoufie, M. (2013) urban growth and transport infrastructure interaction in jeddah between 1980 and 2007.
15	Road transport technologies	Streimikiene, D. (2013) comparative assessment of road transport technologies.
16	Peak travel	Garceau, T.J. (2014) peak travel and the decoupling of vehicle travel from the economy a synthesis of the literature.
17	Urban surrounding	Chatziioannou, I. (2017) evaluation of the urban transportation infrastructure and its urban surroundings in the iztapalapa county: a geotechnology approach about its management.
18	biomass-derived acid	Serrano-Ruiz, J.C. (2012) catalytic transformations of biomass-derived acids into advanced biofuels.
19	Transport infrastructure development	De Jong, M. (2012) the pros and cons of confucian values in transport infrastructure development in China.
20	transport infrastructure slope	Smethurst, J.A. (2017) current and future role of instrumentation and monitoring in the performance of transport infrastructure slopes.

## Appendix C. High-Impact Number in Top 10 Clusters

**Clusters**	**Year**	**Authors**	**Clusters**	**Year**	**Authors**
#0 Cost overrun	2008	Priemus H	#3 Local development	2008	Weisbrod G
		Banister D			Crescenzi R
		Khadaroo J			Lopez E
	2010	Dimitriou HT		2010	Lopez E
		Tang LY			Brocker J
		Cantarelli CC			Gutierrez J
	2012	Beukers E		2012	Nensher DA
		Cantarelli CC			Levinson DM
		Cantarelli CC			Crescenzi R
#1 Quantitative spatial economics	2012	Koopmans C	#4 Land value	2013	Nakagawa S
		Banerjee A			Mchammad SI
		Duranton G			Melo PC
	2007	Baird AJ		2010	Percoco M
		Wagner J			Cohen JP
		Baum-snow N			Munoz-Raskin R
	2014	Kline P		2015	Bensassi S
		Duranton G			Mattsson LG
		Faber B			Reggiani A
#2 Prioritizing highway defragmentation location	2009	Balkenhol N	#5 regional economic	2011	Banister D
		Glista DJ			Hong JJ
		Fahrig L			Lakshmanan TR
	2010	Landguth EL		2009	Jiwattanakulpaisarn P
		Benitez-Lopez A			Bronzini R
		Holderogger R			Lesage J
	2012	Zeller KA		2013	Na ky
		Hepenstrick D			Yu NN
